# Expression Quantitative Trait Loci for CARD8 Contributes to Risk of Two Infection-Related Cancers—Hepatocellular Carcinoma and Cervical Cancer

**DOI:** 10.1371/journal.pone.0132352

**Published:** 2015-07-06

**Authors:** Jian Yin, Juan Wen, Dong Hang, Jing Han, Jie Jiang, Ci Song, Yao Liu, Jibin Liu, Li Liu, Liguo Zhu, Jianguo Chen, Xiangjun Zhai, Shuanghua Xie, Zhibin Hu, Hongbing Shen, Min Dai, Ni Li

**Affiliations:** 1 Department of Epidemiology and Biostatistics, Jiangsu Key Lab of Cancer Biomarkers, Prevention and Treatment, Collaborative Innovation Center For Cancer Personalized Medicine, School of Public Health, Nanjing Medical University, Nanjing, China; 2 Pathology Center and Department of Pathology, Soochow University, Suzhou, China; 3 Department of Hepatobiliary Surgery, Nantong Tumor Hospital, Nantong, China; 4 Digestive Endoscopy Center, the First Affiliated Hospital of Nanjing Medical University, Nanjing, China; 5 Department of Infection Diseases, Jiangsu Province Center for Disease Prevention and Control, Nanjing, China; 6 Qidong Liver Cancer Institute, Qidong, China; 7 National Office for Cancer Prevention and Control, Cancer Institute and Hospital, Chinese Academy of Medical Science, Beijing, China; Medical College of Soochow University, CHINA

## Abstract

Caspase recruitment domain family, member 8 (CARD8) can coordinate innate and adaptive immune responses and sensitize cells to apoptosis, which may participate in tumorigenesis of virus-induced hepatocellular carcinoma (HCC) and cervical cancer. By bioinformatics analyses, we identified several single nucleotide polymorphisms (SNPs) within a new identified long non-coding RNA (lncRNA) as expression quantitative trait loci (eQTLs) for CARD8. In this study, we therefore hypothesized that CARD8 eQTLs SNPs within lncRNA may influence the risk of HCC and cervical cancer. We performed two independent case-control studies of 1,300 cases with HBV-positive HCC and 1,344 normal controls, together with 1,486 cervical cancer patients and 1,536 control subjects to test the association between eQTLs SNP (rs7248320) for CARD8 and the risk of HCC and cervical cancer. The variant genotype of rs7248320 was significantly associated with increased risk of HCC and cervical cancer [GG vs. AA/GA: adjusted odds ratio (OR) = 1.28, 95% confidence interval (CI) = 1.03–1.61, *P* = 0.028 for HCC; adjusted OR = 1.34, 95% CI = 1.09–1.66, *P* = 0.006 for cervical cancer]. Moreover, the effect of rs7248320 on cervical cancer risk was more prominent in premenopausal women. Further interactive analysis detected a significantly multiplicative interaction between rs7248320 and menopausal status on cervical cancer risk (*P* = 0.018). These findings suggest that CARD8 eQTLs SNP may serve as a susceptibility marker for virus-related HCC and cervical cancer.

## Introduction

Hepatocellular carcinoma (HCC) and cervical cancer are two most common cancers throughout the world. In 2008, it was estimated that more than 80% of the HCC and cervical cancer cases occurred in developing countries, including China [1,2]. Persistent Hepatitis B virus (HBV) and human papillomavirus (HPV) infection have been well established as risk factors for liver [3] and cervical cancer [4]. However, only a small fraction of viral persistent carriers eventually develop cancer, suggesting other factors may influence clinical outcomes after virus infections. It was reported that host genetic variations play a critical role in carcinogenesis of HCC and cervical cancer [5,6].

Caspase recruitment domain family, member 8 (CARD8) represents a new member of caspase-associated recruitment domains (CARD) family, which are protein-protein interaction modules found extensively in proteins that play important roles in apoptosis and nuclear factor kappa-B (NFκB) activation [7]. Defects in apoptosis are seen in virtually all types of human cancers [8]. CARD8 can suppress activity of NFκB activated by inflammatory mediators [9]. Moreover, activation of NFκB, a hallmark of inflammatory response, is frequently detected in tumors and may constitute a missing link between inflammation and cancer [10,11], and it is critical in the development of virus-induced cancers including HCC and cervical cancer [11–13]. For example, the activation of NFκB signal transduction requires the interaction between hepatitis B virus X protein and oncogene AIB1 [14], and human papillomavirus 16 E5 oncoprotein mediates cervical carcinogenesis via up-regulation of COX-2 expression through NFκB [11].

Long non-coding RNAs (lncRNAs) are defined as non protein-coding transcripts larger than 200 nucleotides. They control gene expression in the nucleus by modulating transcription or via post-transcriptional mechanisms targeting the splicing, stability, or translation of mRNAs [15]. LncRNA AC008392.1 is one of newly identified lncRNAs, which locates in the upstream region of CARD8 in the nineteenth chromosome and is expressed in multiple cell lines such as GM12878 (Human B lymphocyte) and HeLaS3 (Human cervical cancer cells). By bioinformatics analyses, we identified two single nucleotide polymorphisms (SNPs) (rs7248320 and rs12459322) in AC008392.1 may represent the expression quantitative trait loci (eQTLs) for CARD8 (http://www.regulomedb.org) [16]. It is possible that the SNPs influence the interaction between AC008392.1 and CARD8, thereby altering the expression of CARD8. Here, we hypothesized that CARD8 eQTLs SNPs may contribute to modify the risk of two virus-related HCC and cervical cancer. To test our hypothesis, we conducted two independent case-control studies including 1,300 cases with HBV-positive HCC and 1,344 control subjects, as well as 1,486 cervical cancer cases and 1,536 control subjects to assess the associations between eQTLs SNPs for CARD8 and the susceptibility of the two cancers.

## Materials and Methods

### Study Subjects

This study was approved by the ethics committee of Cancer Institute and Hospital, Chinese Academy of Medical Sciences and Nanjing Medical University. All the participants provide their written informed consent to participate in this study and the ethics committees approved this consent procedure. The subjects’ enrollment was described previously [17–20].

Briefly, HCC patients were consecutively recruited between January 2006 and December 2010 at the Nantong Tumor Hospital, Qidong Liver Cancer Institute, and the First Affiliated Hospital of Nanjing Medical University of Jiangsu Province, China. The diagnosis of HCC was substantiated by a pathological examination and/or alpha-fetoprotein elevation (> 400 ng/mL) combined with magnetic resonance imaging and/or computerized tomography. Because HCV infection is rare in Chinese populations, The HCC patients with HCV infection were excluded. As a result, 1,300 HBV-positive HCC patients consented to participate in the study. From September 2009 to March 2010, 149,172 inhabitants, who were residing in fifty-two villages in Jiangsu province, China for at least a full year, were enrolled in a community-based cross sectional study. Among them, a total of 11,474 participants were HBsAg seropositive. Afterwards, from September to November 2010, 8,006 individuals (69.8%, 8006/11474) who agreed to be regularly followed up every 1–2 years were called back and checked Hepatitis virus seromarkers once again. Finally, 7,250 individuals who were HBsAg seropositive with twice samples collection in at least 6-month interval, negative for HCV and having information of antiviral therapy were enrolled the community-based cohort. Among them, a total of 1,344 subjects were randomly selected as the controls served as a comparison group of HCC cases. All of the controls were HBV persistent carriers who were positive for both HBV surface antigen (HBsAg) and antibody against hepatitis B core antigen (anti-HBc), but negative for HCV antibody (anti-HCV). No history of HBV vaccination was reported for these subjects. These selected controls were matched to the HCC cases on age (± 5 years) and gender, and declared no previous malignancy.

For the cervical cancer study, the 1,486 histologically-confirmed cervical cancer patients were consecutively recruited between March 2006 to December 2010 from the Nantong Tumor Hospital and the First Affiliated Hospital of Nanjing Medical University, Jiangsu, China. The 1,536 controls were also randomly selected from a control pool more than 40,000 individuals who took part in another community-based screening program for non-infectious diseases conducted in Jiangsu Province with follow-up rate of 98.1%. The controls were frequency-matched to the cases on age (± 5 years). All of the controls had no self-reported cancer history.

After written informed consent was obtained, each participant was scheduled for an interview by using a structured questionnaire to collect information on demographic data, menstrual and reproductive history, and environmental exposure history. Individuals who smoked one cigarette per day for more than 1 year were defined as smokers, and those who consumed one or more alcohol drinks per week for more than 6 months were categorized as alcohol drinkers. All patients and controls were unrelated ethnic Han Chinese. After interview, a venous blood sample of approximately 5 ml was collected from each participant (HCC patient, cervical cancer patient and control).

### Serological Testing

HBsAg, anti-HBc and anti-HCV were detected from each participant’s serum by the enzyme-linked immunosorbent assay (Kehua Bio-engineering Co., Ltd., Shanghai, China) following the manufacturer’s instructions as described previously [17].

### SNPs Selection and Genotyping

Based on the data from UCSC database (GRCh37/hg19), Regulome database and the criteria of minor allele frequency (MAF) > 0.05 in Han Chinese, we found two common eQTLs SNPs for CARD8 in nearby lncRNA AC008392.1. The two CARD8 eQTLs SNPs (rs12459322 and rs7248320) are in high linkage disequilibrium (LD) (D’ = 1.000). Thus, we chose rs7248320 as the tagging SNP. Genomic DNA was extracted from a leukocyte pellet by traditional proteinase K digestion and followed by phenol-chloroform extraction and ethanol precipitation. SNP rs7248320 was genotyped using the Sequenom MassARRAY iPLEX platform (Sequenom Inc). The information on primers is shown in Table 1. The genotyping was performed blindly without knowing the status of case or control. Two blank (water) controls in each 384-well plate were used for quality control and more than 10% samples were randomly selected to be repeated, yielding a 100% concordance rate.

**Table 1:** Information of primers for Sequenom MassARRAY Iplex.

SNP	Primer	Sequence (5’-3’)
**rs7248320**	1st-PCR Primer	ACGTTGGATGACAGCCTGGAGGAATCTAAG
	2nd-PCR Primer	ACGTTGGATGTTCTCTTAACGTCCTCTTTC
	Extend Primer	TCTTCTCCAAGATCGAAT

### Statistical Analysis

Differences of demographic characteristics and genotype frequencies of SNP rs7248320 between the cases and controls were calculated by the Student’s t-test (for continuous variables) and χ^2^ test (for categorical variables). Associations between the genotypes and cancer risks were estimated by computing odds ratios (OR) and their 95% confidence intervals (CIs) from logistic regression analyses. Heterogeneity of associations between subgroups was assessed by the χ^2^-based *Q* test. All of the statistical analyses were performed with R software (version 2.13.0; The R Foundation for Statistical Computing). All tests were two-sided and the criterion of statistical significance was set at *P* < 0.05.

## Results

The demographic characteristics of the 1,300 HBV-positive HCC patients and 1,344 control subjects, and 1,486 cervical cancer patients and 1,536 control subjects were described previously (Tables 2 and 3) [17–20]. There were no significant differences in the proportion of age, gender and smoking between the HCC cases and controls. However, the proportion of drinking was significantly higher in HCC cases than in controls (*P* < 0.001). Compared with the control subjects, the cervical cancer patients had significantly higher proportion of smoking (*P* < 0.001), premenopausal status (*P* = 0.002), and higher parity (*P* = 0.001).

**Table 2:** Demographic and selected variables in HCC cases and controls.

Variables	HCC	Controls	*P-*value
	(*n* = 1300) N (%)	(*n* = 1344) N (%)	
**Age, year (mean±SD)**	52.9±10.5	52.7±11.1	0.765
**Age, year**			0.613
**≤53**	693(53.3)	703(52.3)	
**>53**	607(46.7)	641(47.7)	
**Gender**			0.914
**Male**	1104(84.9)	1139(84.7)	
**Female**	196(15.1)	205(15.3)	
**Smoking status**			0.287
**Ever**	774(59.5)	772(57.4)	
**Never**	526(40.5)	572(42.6)	
**Drinking status**			<0.001
**Ever**	764(58.8)	602(44.8)	
**Never**	536(41.2)	742(55.2)	

Abbreviation: SD, standard deviation.

**Table 3:** Demographic and selected variables between cervical cancer cases and controls.

Variables	Cases	Controls	*P-*value
	(*n* = 1486) N(%)	(*n* = 1536) N(%)	
**Age, year (mean±SD)**	53.85±12.70	53.21±11.91	0.159
**Smoking status**			<0.001
**Smoker**	62(4.2)	22(1.4)	
**Non-smoker**	1404(95.8)	1514(98.6)	
**Menopausal status**			0.002
**Premenopausal**	608(41.5)	598(38.9)	
**Natural menopause**	769(52.5)	878(57.2)	
**Unnatural menopause**	81(5.5)	60(3.9)	
**Family history of any cancer**			0.335
**Yes**	278(19.0)	313(20.4)	
**No**	1187(81.0)	1223(79.6)	
**Parity**			0.001
**0~1**	620(42.3)	731(48.3)	
**2**	406(27.7)	405(26.8)	
**>2**	440(30.0)	377(24.9)	

Abbreviation: SD, standard deviation.

The genotype distributions of rs7248320 in the patients and controls were shown in Table 4. The genotype frequencies of rs7248320 were in Hardy-Weinberg equilibrium in the controls (*P* = 0.718 for HCC controls and *P* = 0.343 for cervical cancer controls). In the multivariate logistic regression models, rs7248320 variant genotype GG was associated with an increased risk in HCC by 1.28-fold (95% CI = 1.03–1.61, *P* = 0.028) compared with the genotypes AA/GA. Similarly, the GG genotype increased the risk of cervical cancer by 1.34-fold (95% CI = 1.09–1.66, *P* = 0.006) when compared with the genotypes AA/GA (Table 4).

**Table 4:** Logistic regression analyses on associations between single nucleotide polymorphism rs7248320 (A>G) genotypes and risk of hepatocellular carcinoma and cervical cancer.

Genotype	Hepatocellular carcinoma						Cervical cancer					
	Patients (N = 1300) *n* (%)	Controls (N = 1344) *n* (%)	OR(95%CI)	*P*	OR(95%CI)^a^	*P* ^a^	Patients (N = 1486) *n* (%)	Controls (N = 1536) *n* (%)	OR(95%CI)	*P*	OR(95%CI)^b^	*P* ^b^
**rs7248320**												
**AA**	524(41.3%)	564(42.4%)	1		1		535(39.5%)	614(42.3%)	1		1	
**GA**	540(42.5%)	600(45.1%)	0.97(0.82–1.14)	0.708	0.97(0.82–1.15)	0.755	587(43.4%)	640(44.1%)	1.03(0.88–1.20)	0.706	1.05(0.89–1.24)	0.519
**GG**	206(16.2%)	167(12.5%)	1.33(1.05–1.68)	**0.019**	1.27(1.00–1.61)	0.053	231(17.1%)	196(13.5%)	1.37(1.10–1.70)	**0.005**	1.38(1.10–1.73)	**0.005**
**GG vs. AA/GA**			1.35(1.08–1.68)	**0.008**	1.28(1.03–1.61)	**0.028**			1.35(1.10–1.65)	**0.004**	1.34(1.09–1.66)	**0.006**

CI, confidence interval; OR, odds ratio. The boldface values represent these *P* values were less than 0.05.

^a^Adjusted for age, gender, smoking status and drinking status.

^b^Adjusted for age, smoking status, menopausal status, family history of any cancer and parity.

The association between rs7248320 and the risk of HCC was further examined in stratified analysis (Table 5). In recessive model, the risk effect for rs7248320 was slightly more prominent in younger subjects (≤ 53 years), male subjects, and those who had ever smoked. However, heterogeneity test was not significant for these strata. Table 6 displayed the association of rs7248320 with the risk of cervical cancer. Although the risk effect for rs7248320 was slightly more pronounced among younger women (≤ 53 years), subjects who had never smoked, subjects who had no family history of any cancer, and less parity (0~1), no heterogeneity between these strata was observed. Interestingly, a stronger risk effect of rs7248320 was observed among premenopausal women (adjusted OR = 1.82, 95% CI = 1.33–2.48) (*P* = 0.014 for heterogeneity test). By further interactive analysis, a significantly multiplicative interaction was observed between rs7248320 and menopausal status for the risk of cervical cancer (*P* = 0.018). The crossover analysis suggested that compared with menopause women with genotypes AA/GA, premenopausal women with genotype GG had the highest risk for cervical cancer (adjusted OR = 3.18, 95% CI = 2.24–4.52, *P* < 0.001) (Table 7).

**Table 5:** Stratified analyses on association between rs7248320 genotypes and HCC risk.

Variables	Patients/Controls N (%)		OR(95%CI)^a^	*P* ^b^
	AA/GA	GG		
**Age**				0.280
**≤53**	560(82.7)/607(87.6)	117(17.3)/86(12.4)	1.41(1.04–1.92)	
**>53**	504(85.0)/557(87.3)	89(15.0)/81(12.7)	1.10(0.79–1.53)	
**Gender**				0.133
**Male**	900(83.0)/985(87.3)	184(17.0)/143(12.7)	1.36(1.07–1.73)	
**Female**	164(88.2)/179(88.2)	22(11.8)/24(11.8)	0.77(0.38–1.55)	
**Smoking status**				0.625
**Ever**	617(81.4)/657(86.2)	141(18.6)/105(13.8)	1.35(1.01–1.80)	
**Never**	447(87.3)/507(89.1)	65(12.7)/62(10.9)	1.20(0.83–1.75)	
**Drinking status**				0.922
**Ever**	607(81.4)/505(84.9)	139(18.6)/90(15.1)	1.29(0.96–1.73)	
**Never**	457(87.2)/659(89.5)	67(12.8)/77(10.5)	1.32(0.92–1.88)	

^a^Adjusted for age, gender, smoking status and drinking status (excluded the stratified factor in each stratum).

^b^ *P*-value for the heterogeneity test.

**Table 6:** Stratified analyses on association between rs7248320 genotypes and cervical cancer risk.

Variables	Patients/ Controls N (%)		OR(95%CI)^a^	*P* ^b^
	AA/GA	GG		
**Age**				0.118
**≤53**	554(79.5)/679(85.3)	143(20.5)/117(14.7)	1.59(1.21–2.10)	
**>53**	567(86.6)/575(87.9)	88(13.4)/79(12.1)	1.13(0.81–1.56)	
**Smoking status**				0.498
**Ever**	45(86.5)/20(90.9)	7(13.5)/2(9.1)	2.49(0.42–14.79)	
**Never**	1076(82.8)/1234(86.4)	224(17.2)/194(13.6)	1.34(1.09–1.66)	
**Menopausal status**				0.014
**Premenopausal**	470(78.9)/508(86.5)	126(21.1)/79(13.5)	1.82(1.33–2.48)	
**Menopause**	651(86.1)/746(86.4)	105(13.9)/117(13.6)	1.07(0.80–1.42)	
**Family history of any cancer**				0.830
**Yes**	210(82.7)/256(86.2)	44(17.3)/41(13.8)	1.41(0.87–2.28)	
**No**	911(83.0)/998(86.6)	187(17.0)/155(13.4)	1.33(1.06–1.68)	
**Parity**				0.305
**0–1**	455(80.2)/612(86.3)	112(19.8)/97(13.7)	1.6(1.19–2.16)	
**2**	319(83.5)/336(85.1)	63(16.5)/59(14.9)	1.09(0.73–1.62)	
**>2**	347(86.1)/306(88.4)	56(13.9)/40(11.6)	1.29(0.83–2.00)	

^a^Adjusted for age, smoking status, menopausal status, family history of any cancer and parity (excluded the stratified factor in each stratum).

^b^ *P*-value for the heterogeneity test.

**Table 7:** Crossover analysis in genotypes-menopausal status interaction study on cervical cancer.

Genotype	Menopausal status	Patients (N = 1486) *n* (%)	Controls (N = 1536) *n* (%)	OR(95%CI)^a^	*P* ^*a*^
**AA/GA**	Menopause	642(48.15)	759(51.53)	1	
**AA/GA**	Premenopausal	471(34.79)	517(35.10)	1.80(1.42–2.29)	<0.001
**GG**	Menopause	105(7.75)	117(7.94)	1.06(0.79–1.41)	0.700
**GG**	Premenopausal	126(9.31)	80(5.43)	3.18(2.24–4.52)	<0.001
**Interaction**				*P* ^b^ = 0.018	

^a^Adjusted for age, smoking status, family history of any cancer and parity.

^b^ *P* value for multiplicative interaction.

## Discussion

In these two independent case-control studies, we investigated the association between rs7248320 and the susceptibility of HCC and cervical cancer from a Chinese population. We found that eQTLs SNP rs7248320 for CARD8 was associated with the increased risk of HCC and cervical cancer. Moreover, a significantly multiplicative interaction between rs7248320 and menopausal status for the risk of cervical cancer was also observed.

Since the ENCODE Project and RNA-seq analysis have identified thousands of new lncRNAs, the genetic variants and biological function of lncRNAs are becoming hot spot in studies of complex diseases. Several studies reported that multiple lncRNAs were dysregulated in HCC and cervical cancer, such as HOTAIR and MEG3 [21–25]. Besides, we previously reported that ZNRD1 eQTLs SNPs increased the risk of HCC [20]. LncRNA AC008392.1 locates in the upstream region of CARD8, which may influence the expression of CARD8. A previous study showed that the variant genotype of rs7248320 and rs12459322 were associated with the down-expression of CARD8 in monocytes [26]. There is evidence to suggest that CARD8 is a negative regulator of NFκB activation, which results in the suppression of immune and inflammatory [27,28]. In addition, the reduction of CARD8 suggests a decrease in anti-apoptotic defense [29]. The activation of NFκB signaling contributes to carcinogenesis and promotes disease prognosis and metastasis in HCC and cervical cancer [30–34]. Thus, the variant genotype GG of rs7248320 in lncRNA AC008392.1 may be associated with activation of NFκB and apoptosis inhibition through down-regulation of CARD8, and consequently promote the development and progression of infection-related cancer, including HCC and cervical cancer, which was consistent with the risk effect of the variant genotype GG of rs7248320 in the results of our study.

There are several limitations of the study which need to be solved in the further research. Firstly, we conducted the case-control study for only one stage without validation. Further large-scale studies are required for validating the associations between rs7248320 in AC008392.1 and the risk of HCC and cervical cancer. Secondly, the biological function of the two eQTLs SNPs at AC008392.1 was not further investigated in this study. The associations between the SNPs and the risk of HCC and cervical cancer should be interpreted with caution. While, in our previous study [20], We found ZNRD1 (Zinc ribbon domain containing 1) eQTLs SNP rs3757328 in ZNRD1-AS1 (ZNRD1 antisense RNA 1) was associated with increased risk for HCC, and further eQTLs analysis results indicated the significantly association between the genotypes of rs3757328 and the expression of ZNRD1 and ZNRD1-AS1. In vitro experiments also showed that ZNRD1 knockdown inhibited the expression of HBV mRNA and promoted proliferation of HepG2.2.15 cells. Given that the findings from our previous study [20], we hypothesized that lncRNA AC008392.1 might regulate the expression of related protein (CARD8) by itself variation and thereby influence the occurrence and development of hepatic tumor and cervical tumor. Thirdly, HPV infection, the primary and necessary cause of cervical cancer, was not detected in the cervical specimens. And the interaction between HPV type and rs7248320 could not be assessed in cervical carcinogenesis.

To our knowledge, this is the first study investigating the association of CARD8 eQTLs SNPs in the lncRNA AC008392.1 with the risk of HCC and cervical cancer. With a relatively large population, this study revealed that the variant genotype GG of rs7248320 in lncRNA AC008392.1 influenced the risk of two virus-related cancers—HCC and cervical cancer. The variant genotype GG combined with premenopausal status strengthened the risk effect. These results suggested that AC008392.1 rs7248320 might serve as a susceptibility marker for HCC and cervical cancer. Further studies incorporating diverse populations and functional assays are warranted to validate and extend our findings.
